# Supplementary material for: Heterogeneous and short-term effects of a changing climate on farmers’ labor allocation: An empirical analysis of China
Source: PLoS One. 2024 Jul 26;19(7):e0306260. doi: 10.1371/journal.pone.0306260 (PMC11280529; doi:10.1371/journal.pone.0306260)
Supplement: Attachment — Submitted filename: renamed_2f9a3.docx [file pone.0306260.s002.docx]

**Farmers’ Labor Allocation Response to Climate Change in China**

**Comments**

The message and idea of the paper is very appealing. The article requires significant revisions to meet the publication standards of Journal and my observations are as follows:

1. Firstly, the title needs improvement to better represent the study's purpose.
2. The introduction section appears to be somewhat weak and could benefit from further elaboration. It is essential to highlight the importance of the subject area and provide a clear understanding of the problem. Additionally, including a theoretical framework for the study is recommended. Moreover, I suggest adding more relevant literature and citations to strengthen the introduction.
3. There is a significant need to more elaborate the discussion section in the paper with latest references.
4. The conclusion section could benefit from the addition of information regarding limitations and suggestions for future research.
5. In general, I found the manuscript interesting, but it seems somewhat simplistic in its current form. Authors should incorporate statistical techniques and include more citations to enhance the paper's quality.
